# Supplementary material for: Differences in referral path, clinical and radiographic outcomes between seronegative and seropositive rheumatoid arthritis Mexican Mestizo patients: A cohort study
Source: PLoS One. 2024 Jun 6;19(6):e0304953. doi: 10.1371/journal.pone.0304953 (PMC11156376; doi:10.1371/journal.pone.0304953)
Supplement: Attachment — Submitted filename: RESPONSES TO EDITOR AND REVIEWERS.docx [file pone.0304953.s004.docx]

**RESPONSES TO EDITOR AND REVIEWERS**

**Reviewer #1: Dear Editor:**

I have reviewed the corrected version of the manuscript now titled “Differences in referral path, clinical and radiographic outcomes between seronegative and seropositive rheumatoid arthritis Hispanic patients: A cohort study.” and I consider that the authors have carried out, from my point of view, the suggested changes, or have considered not making changes based on well-founded arguments, so I consider that the manuscript could be eligible for acceptance by your journal. However, the conditionality of acceptance would be subject to making some minor changes that, from my point of view, would increase the scientific quality of the content of the manuscript.

Since these changes are minor, I consider that the manuscript could be accepted without further revision on my part, if you consider that these minimal changes were carried out by the authors.

These changes are:

Consider whether the ethnic background of the target population is more accurately described under the term “Mexican Mestizo,” rather than “Hispanic,” both in the title and throughout the content.

***Response. We have adopted the suggestion.***

There are small discrepancies in the percentage frequencies of patients who developed erosive disease in the summary, in the text of the results, in the corresponding table, and in the discussion. These percentage frequencies must be unified.

***Response. We apologize for the mistake and have unified the numbers along the text.***

Correct the term of the epidemiological indicator for the percentage frequencies of erosive disease. At baseline, the term “prevalence” is appropriate, but from the first year onwards, given that it is the result of outcomes that occur throughout the follow-up, the appropriate term, from my point of view, is incidence.

***Response. We have adopted the suggestion.***

In the introduction (page 14 of manuscript, line 88), I consider that the most appropriate term is “Caucasian” instead of “White population”.

***Response. We have adopted the suggestion.***

Consider changing the location of Table 1 from the Materials and Methods section to the Results section, since these are actually baseline results.

***Response. We have adopted the suggestion.***

In the first line of the “Radiographic outcomes” section (Table 3) of the Results, I consider that it should be specified that the erosive disease was detected only in seropositive patients, both at baseline and throughout follow-up. Additionally, the final phrase of this section that says, “Also, more SPPs had erosions compared to SNPs, and these differences reached statistical significance since the first years of follow-up and at the feet location.” may be repetitive, since from the first line of this paragraph it was specified that erosions were only detected in seropositive patients.

***Response. We have updated the paragraph to adopt the suggestions.***

In the heading of Table 4, I consider that the adjective “incidental” should be added to the term “erosive disease”, since this outcome is what this table specifically describes.

***Response. We have adopted the suggestion.***
